# 626. The Efficacy and Safety of Maintenance with Doravirine Plus Two NRTIs after Initial Suppression in Adults with HIV-1 in the DRIVE-FORWARD Clinical Trial: Results from the Study Extension through 192 Weeks

**DOI:** 10.1093/ofid/ofab466.824

**Published:** 2021-12-04

**Authors:** Pedro Cahn, Jean-michel Molina, Johan Lombaard, Kathleen Squires, Sushma Kumar, Hong Wan, Valerie Teal, Ernest Asante-Appiah, Peter Sklar, Elizabeth A Martin, Rima Lahoulou

**Affiliations:** 1 Fundación Huésped, Buenos Aires, Buenos Aires, Argentina; 2 Hopital Saint-Louis, Paris, Ile-de-France, France; 3 JOSHA Research, Bloemfontein, Free State, South Africa; 4 Merck & Co,. Inc, Kenilworth, New Jersey; 5 Merck & Co., Inc., Kenilworth, New Jersey; 6 MSD, Puteaux, Haute-Normandie, France

## Abstract

**Background:**

DRIVE-FORWARD is a phase 3 trial with a completed double-blind period comparing doravirine (DOR) 100 mg with ritonavir-boosted darunavir (DRV/r) 800/100 mg, both administered with two nucleos(t)ide reverse transcriptase inhibitors (NRTIs; tenofovir and emtricitabine, or abacavir and lamivudine), and an ongoing open-label extension. At Week (W) 48, DOR demonstrated non-inferior efficacy to DRV/r, with a superior lipid profile. Those results were sustained at W96. Here we present efficacy and safety results through W192.

**Methods:**

Participants who completed the 96-week double-blind phase and met inclusion criteria were eligible to receive open-label DOR plus two NRTIs in a 96-week extension. Efficacy and safety at W192 were assessed in two groups: participants initially randomized to DOR and maintained on DOR (n=259) and those who switched from DRV/r to DOR at W96 (n=233).

**Results:**

HIV-1 RNA < 50 copies/mL were maintained through W192 in 81.1% of participants who continued DOR and 80.7% of those who switched from DRV/r to DOR. The mean increase in CD4 T-cell counts from W96 to W192 was similar for participants maintained on DOR (47 cells/mm^3^) and those switched from DRV/r (53 cells/mm^3^). Protocol-defined virologic failure occurred in 3.1% and 5.6% of participants maintained on DOR and switched from DRV/r, respectively, and development of genotypic resistance was low in both groups (Table 1). Discontinuation due to adverse events was also low (Table 1). Fasting LDL-cholesterol, non-HDL-cholesterol, and triglycerides showed minimal increase in participants maintained on DOR and were reduced in those switched from DRV/r to DOR (Table 1). Participants maintained on DOR had minimal weight gain after W96 (median 1 kg), and a small increase overall (median 1.9 kg, Day 1 through W192); participants who switched to DOR had a small increase after W96 (median 1.5 kg), similar to the median weight gain in the base study (DOR 1.8 kg; DRV/r 0.7 kg).

**Conclusion:**

Among participants who continued DOR in the DRIVE-FORWARD open-label extension, virologic suppression and favorable safety were maintained for an additional 96 weeks. Participants who switched from DRV/r to DOR maintained virologic suppression and demonstrated favorable safety for 96 weeks.

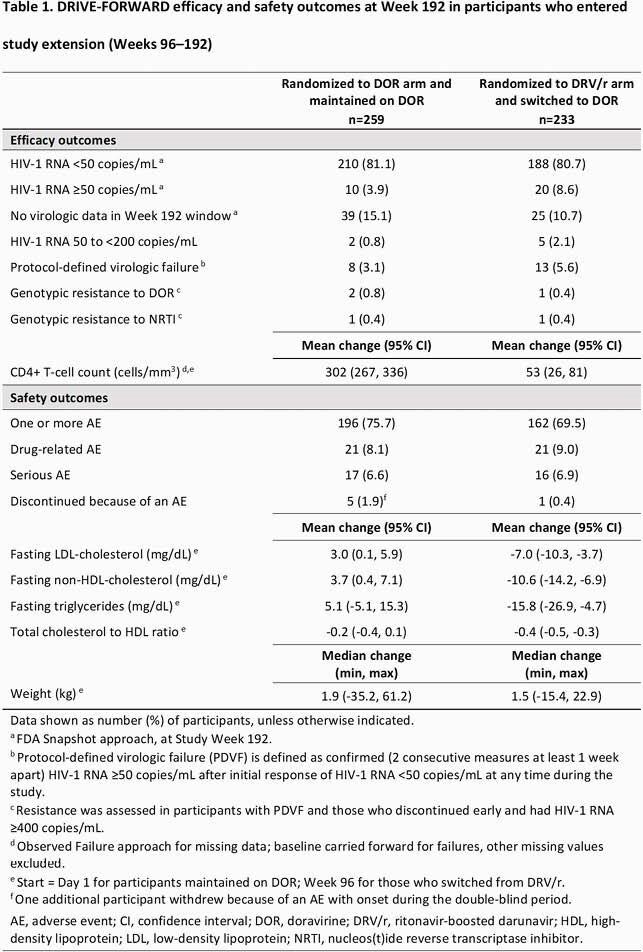

**Disclosures:**

**Pedro Cahn, MD, PHD**, **Merck** (Advisor or Review Panel member)**ViiV Healthcare** (Grant/Research Support, Advisor or Review Panel member) **Kathleen Squires, MD**, **Merck** (Employee) **Sushma Kumar, PhD**, **Merck** (Employee) **Hong Wan, PhD**, **Merck** (Employee) **Valerie Teal, MS**, **Merck** (Employee) **Ernest Asante-Appiah, PhD**, **Merck** (Employee) **Peter Sklar, MD**, **Merck** (Employee) **Elizabeth A. Martin, DO, MPH, MBA**, **Merck** (Employee) **Rima Lahoulou, n/a**, **Merck** (Employee)

